# Comparative Effectiveness of Complete Revascularization Strategies in Patients With ST-Segment Elevation Myocardial Infarction and Multivessel Disease: A Bayesian Network Meta-Analysis

**DOI:** 10.3389/fcvm.2021.724274

**Published:** 2021-09-23

**Authors:** Lingyue Zhao, Wenqin Guo, Weichao Huang, Lili Wang, Fanrui Mo, Xiehui Chen, Chaoyang Li, Siquan Huang

**Affiliations:** ^1^Huazhong University of Science and Technology Union Shenzhen Hospital, Shenzhen, China; ^2^Department of Cardiology, Fuwai Hospital Chinese Academy of Medical Sciences, Shenzhen, China; ^3^Department of Cardiology, The Fourth Affiliated Hospital of Guangxi Medical University, Liuzhou, China; ^4^Department of Cardiology, Shenzhen Longhua District Central Hospital, Shenzhen, China; ^5^People's Hospital of Longhua District, Shenzhen, China

**Keywords:** complete revascularization, ST-segment elevation myocardial infarction, multivessel disease, meta-analysis, randomized controlled trials

## Abstract

Whether fractional flow reserve (FFR) should be available for revascularization in patients with ST-segment elevation myocardial infarction (STEMI) and multivessel disease (MVD) is controversial. We aimed to compare the efficacy of various complete revascularization (CR) regimens for STEMI patients with MVD. The PubMed and Cochrane Library databases and clinicaltrial.gov were searched for the randomized controlled trials (RCTs) comparing the FFR-guided CR, angiography-guided CR, and culprit-only revascularization (COR) strategies in STEMI patients with MVD. A Bayesian random-effect model was employed to synthesize the evidence in network meta-analysis. We used relative risk (RR) and 95% credible interval (CrI) as measures of effect size. The primary endpoint was the composite outcome of all-cause mortality or myocardial infarction (MI). Twelve RCTs were included. Angiography-guided CR showed a lower event rate of the composite outcome (RR, 0.68; 95%CrI, 0.50–0.87), all-cause mortality (RR, 0.75; 95%CrI, 0.55–0.96), MI (RR, 0.63; 95%CrI, 0.43–0.86), and repeat revascularization (RR, 0.36; 95% CrI, 0.24–0.55) compared with COR. Additionally, angiography-guided CR had a lower risk of primary outcome (RR, 0.64; 95%CrI, 0.38–0.94) and MI (RR, 0.58; 95%CrI, 0.31–0.92) than FFR-guided CR. The difference between the FFR-guided CR and COR in terms of composite outcome, all-cause mortality, and MI was similar. Angiography-guided CR was associated with the highest probability of optimal treatment for the primary outcome (98.5%), followed by FFR-guided CR (1.2%) and COR (0.3%). STEMI patients with MVD benefitted more from angiography-guided CR than from FFR-guided CR. However, only one study compared the effectiveness of FFR-guided and angiography-guided PCI; thus, the comparison between FFR-guided and angiography-guided PCI relied on indirect evidence. Therefore, further studies directly comparing the effectiveness of these two CR strategies are warranted.

## Introduction

Approximately 50% of ST-segment elevation myocardial infarction (STEMI) patients are detected with more than one non-culprit vessel with obvious stenosis during coronary angiography (1). STEMI patients with multivessel disease (MVD) have poorer prognosis than those without non-culprit lesions (1, 2). Accordingly, research for optimal revascularization strategies in patients with STEMI and MVD is particularly essential.

Previous evidence has shown that patients receiving complete revascularization (CR), primary intervention to culprit coronary artery, and immediate or staged revascularization to non-culprit artery have decreased incidence of adverse outcomes (e.g., cardiac death and myocardial infarction [MI]) than those receiving culprit-only revascularization (COR) (3, 4). Recently, studies have been conducted to assess the effectiveness of the fractional flow reserve (FFR) technology to guide interventions for those with STEMI and MVD (5, 6). Although FFR-guided CR showed a lower event rate of main adverse cardiovascular events than COR; this benefit was mainly driven by a reduction of repeat revascularization risk but not by reduced adverse outcomes (7). Further, the FLOWER-MI study, the only randomized controlled trial (RCT) comparing the effectiveness between FFR-guided CR and traditional angiography-guided CR, showed that both techniques were effective in reducing the composite outcome of all-cause mortality or MI (8). Whether FFR should be available for revascularization in patients with STEMI and MVD is controversial. Nevertheless, the FLOWER-MI trial had limited statistical power to evaluate the primary outcome due to its small sample size.

A network meta-analysis, which comprehensively synthesizes direct and indirect outcomes, could obtain precise outcomes compared with the outcomes from direct evidence (9, 10). Accordingly, the purpose of our study was to perform a network meta-analysis to compare the effectiveness between FFR-guided and angiography-guided CRs in those with STEMI and MVD.

## Methods

We reported the research based on the Preferred Reporting Items for Systematic Reviews and Meta-Analyses Statement (11).

### Search Strategy and Information Sources

We searched the PubMed and Cochrane Library databases, clinicaltrial.gov, and the references of relevant articles published between January 1, 2000 and May 19, 2021. We employed the following keywords and Medical Subject Headings: “acute ST-segment elevated myocardial infarction,” “multivessel diseases,” and “percutaneous coronary intervention.”

### Inclusion and Exclusion Criteria

We included studies according to the following criteria: (1) studies including patients with STEMI and MVD; (2) studies including a comparison between the FFR-guided CR, angiography-guided CR, and COR; (3) RCTs; and (4) studies published in English. The exclusion criteria were as follows: (1) studies including patients with STEMI and chronic occlusive disease; (2) studies comparing immediate CR and staged CR; and (3) non-RCTs, such as cohort and observational studies. CR was defined as percutaneous coronary intervention (PCI) in the infarct artery, followed by additional PCI in the non-culprit vessel. If there were multiple different reports from the same trial, we extracted the data from the most recently published report.

### Clinical Outcomes

The composite outcome of all-cause mortality or MI was the primary endpoint. The secondary endpoints included all-cause mortality, repeat revascularization, and MI. The above endpoints were defined based on the definitions used in each trial.

### Data Extraction and Study Quality Assessment

Two researchers independently extracted the contents from the included studies: year of publication, follow-up time, revascularization strategy, definition of MVD, population characteristics (e.g., average age, prevalence of diabetes mellitus, and MI), the events of outcome, and the total number of patients in each group. When the contents extracted by these researchers were distinct, the third researcher made the decision. We used the Cochrane risk-of-bias tool to evaluate the quality of studies (3).

### Statistical Analysis

In the traditional meta-analysis, relative risk (RR) and the corresponding 95% confidence interval (CI) were used as measures of estimated effect size. To consider unexplained heterogeneity, a random-effect model with the DerSimonian-Laird method was used to synthesize the evidence (12). We used Cochrane *Q* tests and the inconsistency index (*I*^2^ test) to assess heterogeneity between the included studies (13). An *I*^2^ value <25%, 25–50%, 50–75%, and >75% indicated no, low, moderate, and high heterogeneities, respectively (13). The traditional meta-analysis was conducted using STATA Software version 13.0 (StataCorp LP, College Station, TX, USA).

In the network meta-analysis, a random-effect model was employed to completely preserve randomized treatment comparisons among the studies (14). The empirical (log-normal) priors on the variance were employed to produce the posterior distributions of model parameters and fitted four chains with 1200,000 iterations. We applied the Markov chain Monte Carlo method in the analysis (15) and applied the residual deviance to assess the model fit (14). We employed the Brooks–Gelman–Rubin diagnostic to evaluate the convergence (16). The “node-splitting” approach was used to assess the inconsistency between direct and indirect results (17). A Bayesian *p*-value lower than 0.05 indicated inconsistency between direct and indirect evidence. Additionally, we calculated the corresponding probability of being the optimal option for each treatment (18). We conducted Bayesian meta-regression to explore the association between the event rates of the primary outcome and the variables (for example, follow-up time, age, proportion of males, diabetes mellitus, three-vessel disease, and stenosis of the non-culprit vessel) (19). We performed the sensitivity analysis by excluding the unpublished study (PRAGUE 13 study) or using the odd ratio as effect size. Additionally, the comparison-adjusted funnel plot was drawn to evaluate the publication bias. The RR and 95% credible interval (CrI) were employed as measures of effect size. The network meta-analysis was performed by using gemtc and rjags packages in R software (version 3.3.2; R Foundation for Statistical Computing).

## Results

**Figure 1:**
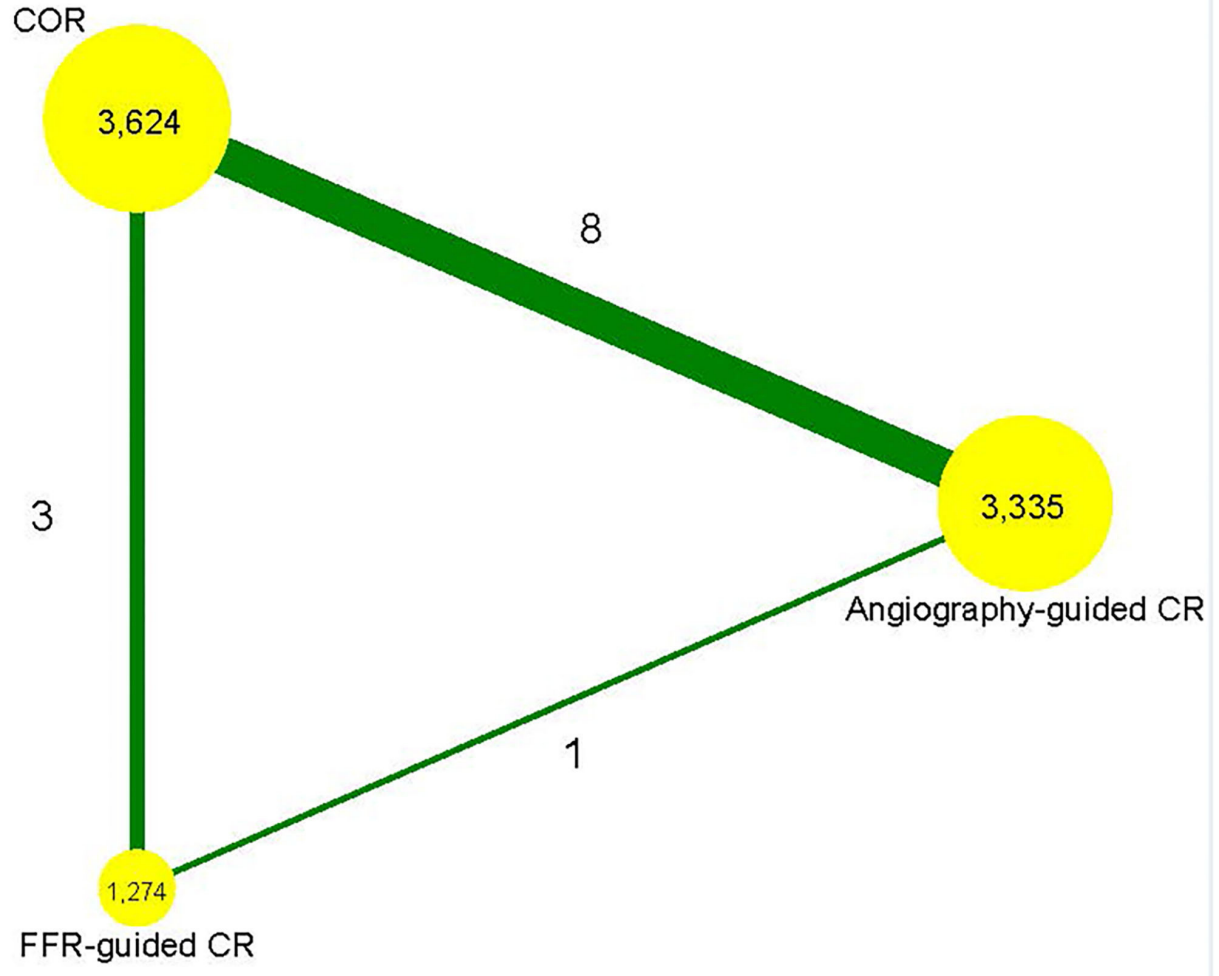
Network plot. FFR, fractional flow reserve; CR, complete revascularization; COR, culprit-only revascularization.

Supplementary Figure 1 shows a flowchart of the research screening process. Initially, 623 studies were screened based on the abstracts. A cohort study conducted by Maamoun et al. was excluded (20). Another study by Ijsselmuiden et al. was excluded since the included patients were without STEMI (21). The PRIMA trial and the study by Terosav et al. were excluded because they compared the effectiveness of immediate and staged revascularization (22, 23), while the EXPLORE study was excluded because it included patients with chronic total occlusion (24). Further, we excluded studies that were short-term reports of a trial (25–27). Finally, 12 randomized controlled trials involving 8,233 patients were included in the analysis (5, 6, 8, 28–36). The network plot in terms of primary outcome is shown in Figure 1. Eight studies compared angiography-guided CR with COR (28, 30–36), three studies compared FFR-guided CR with COR (5, 6, 29), and one study compared FFR-guided CR with angiography-guided CR (8). Table 1 displays the characteristics of these studies. The stenosis of the non-infarct vessel was >70%, following the definition of MVD, in patients included in six of the included studies (30, 32–36), while the other six studies defined MVD as the stenosis of the non-infarct vessel >50% (5, 6, 8, 28, 29, 31). The included studies had a follow-up that ranged from 6 to 67.2 months. The quality emulation of the studies is shown in Supplementary Figure 2. Overall, the studies were associated with a low risk of bias.

**Table 1:** The characteristic of included studies.

**Study/Author**	**Year**	**Comparison**	**Follow-up** **(month)**	**Type of complete** **revascularization**	**Timing of** **staged-procedure** **(Days)**	**Age** **(year)**	**Male** **(%)**	**DM** **(%)**	**EF** **(%)**	**previous** **MI** **(%)**	**Anterior** **MI** **(%)**	**The stenosis** **of non-culprit** **vessel lesson**	**Three-vessel** **disease**
HELP-AMI	2004	Angiography-guided CR vs. COR	12	Index	Not applicable	64	87	19	49	NA	54	≥50%	35
Politi et al.	2010	Angiography-guided CR vs. COR	30	Index	Not applicable	65	77	19	45	NA	44	≥70%	27
PRAMI	2013	Angiography-guided CR vs. COR	23	Index	Not applicable	62	78	18	NA	8	34	≥50%	46
CvLPRIT	2015	Angiography-guided CR vs. COR	67.2	Index hospitalization	3	65	81	14	46	4	36	≥70%	23
Hamza et al.	2016	Angiography-guided CR vs. COR	6	Index hospitalization	3	54	84	100	46	8	47	≥70%	31
PRAGUE 13	2015	Angiography-guided CR vs. COR	38	Staged	3–40	NA	NA	NA	48	NA	NA	≥70%	NA
COMPLETE	2019	Angiography-guided CR vs. COR	36	Index hospitalization	4	62	80	19	NA	7	NA	≥70%	23
Omar et al.	2017	Angiography-guided CR vs. COR	6	Index	Not applicable	55	83	48	55	NA	48	≥70%	15
Compare-Acute	2017	FFR-guided CR vs. COR	36	Index hospitalization	3	62	77	15	NA	8	35	≥50%	32
Ghani et al.	2012	FFR-guided CR vs. COR	36	Staged	7.5	62	80	6	NA	6	NA	≥50%	23
DANAMI-3—PRIMULTI	2015	FFR-guided CR vs. COR	27	Staged	2	64	81	11	50	7	35	≥50%	31
FLOWER-MI	2021	FFR-guided vs. Angiography-guided CR	12	Index hospitalization	2.6	62	83	16	50	7	32	≥50%	23

*FFR, Fractional Flow Reserve; CR, complete revascularization; COR, culprit-only revascularization; NA, not available; DM, diabetes mellitus; EF, ejection fraction; MI, myocardial infarction*.

### Clinical Outcomes

#### Traditional Meta-Analysis

**Figure 2:**
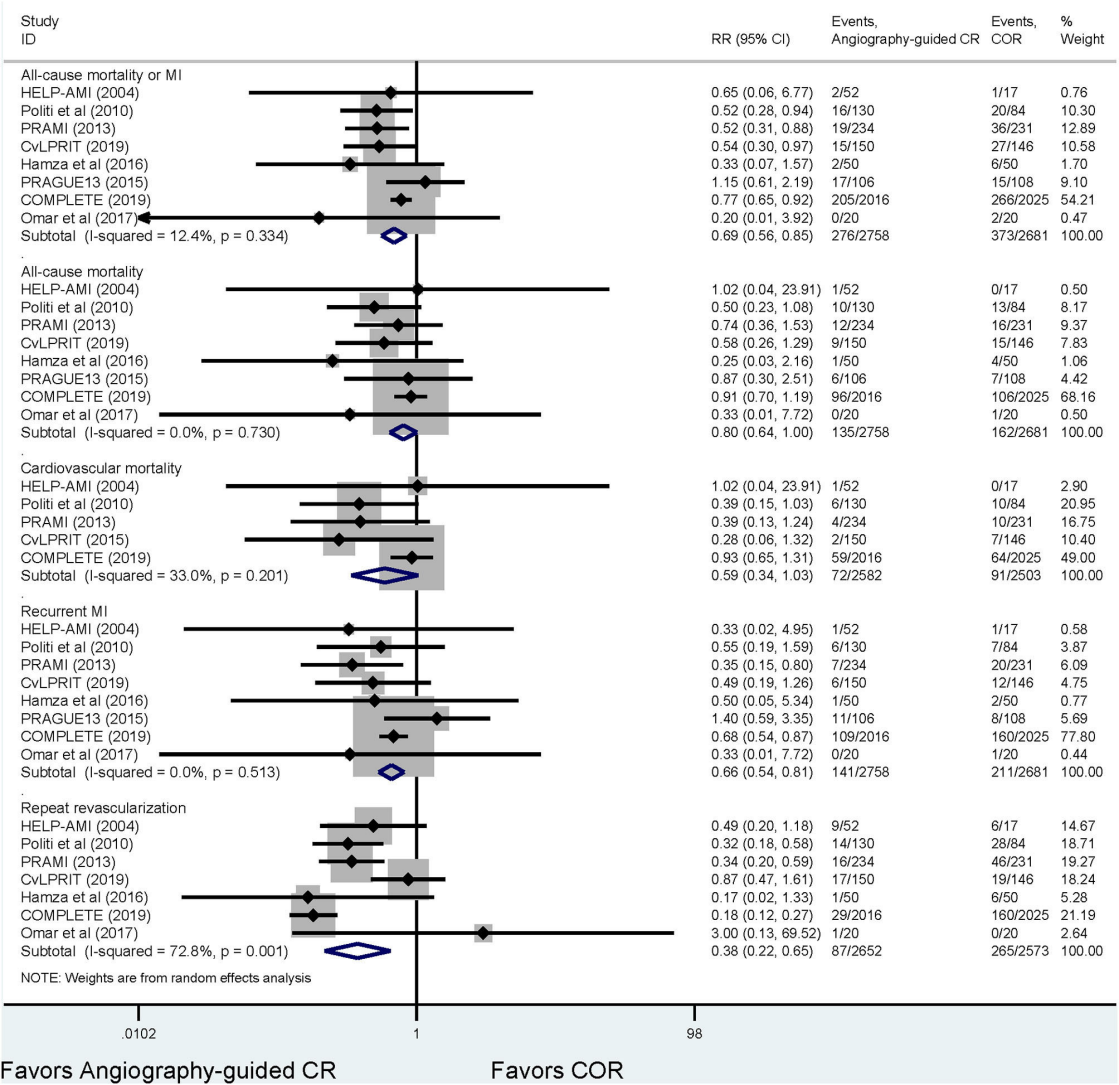
The results of the meta-analysis for the comparison between angiograph-guided complete revascularization and culprit-only revascularization. FFR, fractional flow reserve; COR, culprit-only revascularization; CR, complete revascularization; MI, myocardial infarction.

Figure 2 and Supplementary Figures 3, 4 demonstrate the outcome of the traditional meta-analyses. Angiography-guided CR demonstrated a lower event rate of the primary outcome, MI, and repeat revascularization than COR (RR 0.69, 95% CI 0.56–0.85, *p* = 0.000, *I*^2^ = 12.4% for primary outcome; RR 0.66, 95% CI 0.54–0.81, *p* = 0.000, *I*^2^ = 0 % for MI; and RR 0.38, 95% CI 0.22–0.65, *p* = 0.000, *I*^2^ = 7 2.8% for repeat revascularization, respectively), but the risks of all-cause mortality and cardiovascular mortality were similar between these two treatments (RR 0.80, 95% CI 0.64–1.00, *p* = 0.055, *I*^2^ = 0% for all-cause mortality, RR 0.59, 95% CI 0.34–1.03, *p* = 0.062, *I*^2^ = 33% for cardiovascular mortality, respectively). The event rates of primary outcome, all-cause mortality, cardiovascular mortality, and MI were also similar between FFR-guided PCI and COR (RR 1.07, 95% CI 0.54–2.12, *p* = 0.840, *I*^2^ = 66.0% for primary outcome; RR 1.14, 95% CI 0.67–1.93, *p* = 0.639, *I*^2^ = 0% for all-cause mortality; RR 0.69, 95% CI 0.30–1.62, *p* = 0.400, *I*^2^ = 0% for cardiovascular mortality; and RR 1.01, 95% CI 0.47–2.19, *p* = 0.973, *I*^2^ = 58.2% for MI, respectively); however, FFR-guided PCI indicated a lower repeat revascularization risk than COR (RR 0.54, 95% CI 0.31–0.94, *p* = 0.029, *I*^2^ = 77.6%). The primary outcomes, MI, all-cause mortality, and repeat revascularization did not differ between the angiography-guided CR and FFR-guided CR (RR 0.75, 95% CI 0.43–1.33, *p* = 0.325 for primary outcome; RR 1.13, 95% CI 0.46–2.76, *p* = 0.791 for all-cause mortality; RR 0.56, 95% CI 0.26–1.21, *p* = 0.142 for MI; and RR 0.74, 95% CI 0.35–1.61, *p* = 0.453 for repeat revascularization, respectively).

#### Network Meta-Analysis

**Figure 3:**
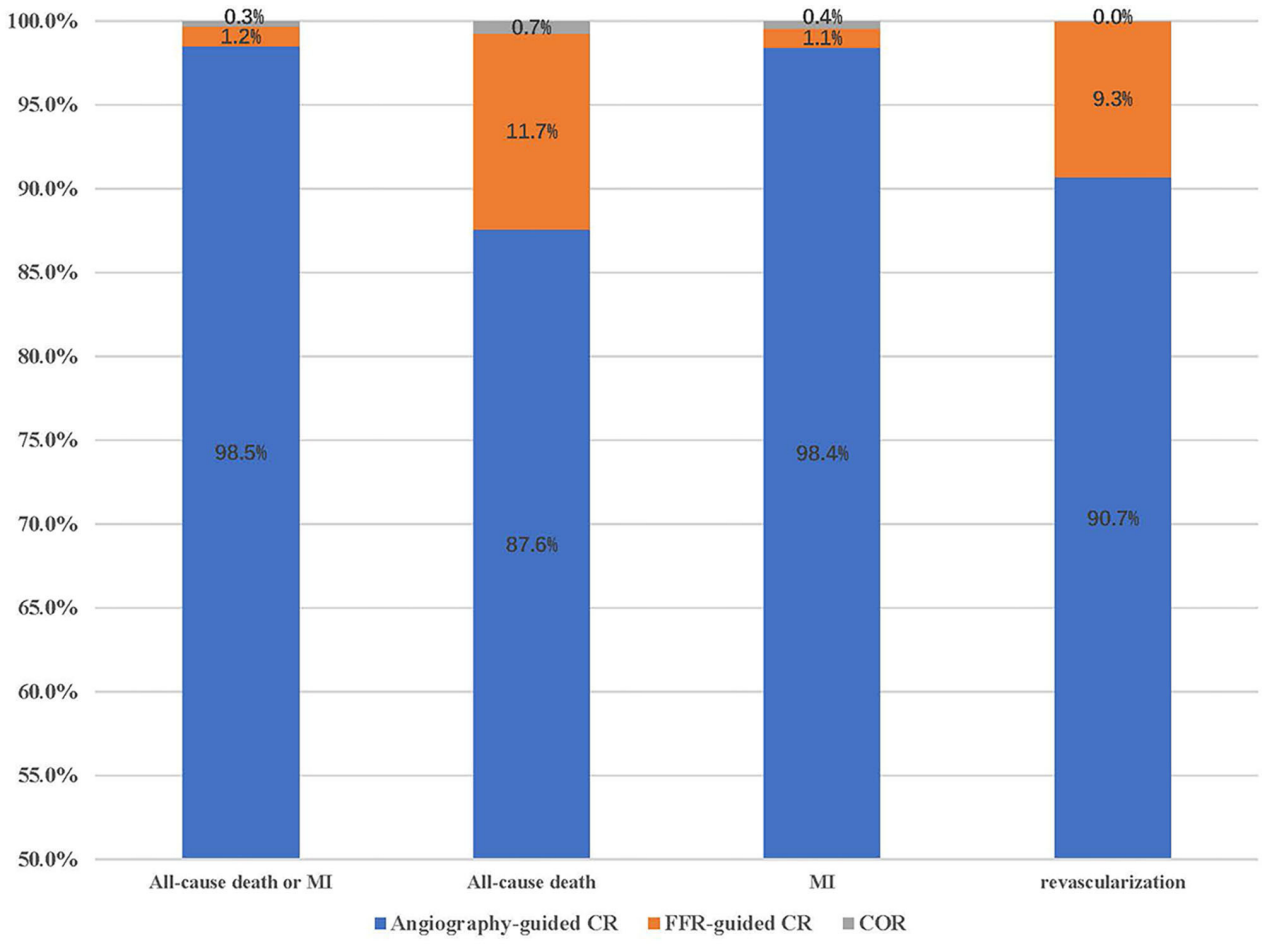
The probability of being the first treatment option for the clinical outcomes. MI, myocardial infarction; COR, culprit-only revascularization.

Table 2 displays the outcomes of Bayesian network meta-analysis. Results showed that angiography-guided CR indicated a lower incidence of the composite outcome, all-cause mortality, MI, and repeat revascularization than COR (RR 0.68 and 95% CrI 0.50–0.87 for primary outcome, RR 0.75 and 95% CrI 0.55–0.96 for all-cause mortality, RR 0.63 and 95% CrI 0.43–0.86 for MI, and RR 0.36 and 95% CrI 0.24–0.55 for repeat revascularization, respectively), but the risk of cardiovascular mortality was similar between these two treatments (RR 0.68 and 95% CrI 0.38–1.01). The risk of occurrence of the composite outcome, all-cause mortality, cardiovascular mortality, and MI was similar between the FFR-guided CR and COR groups (RR 1.06 and 95% CrI 0.75–1.66 for primary outcome, RR 1.03 and 95% CrI 0.62–1.69 for all-cause mortality, RR 0.68 and 95% CrI 0.25–1.71 for cardiovascular mortality, and RR 1.08 and 95% CrI 0.72–1.82 for MI), and the FFR-guided CR indicated a lower repeat revascularization risk than COR (RR 0.53 and 95% CrI 0.32–0.87). Angiography-guided CR indicated a lower incidence of the composite outcome (RR 0.64 and 95% CrI 0.38–0.94) and MI (RR 0.58 and 95% CrI 0.31–0.92) than FFR-guided CR. There was no difference in terms of all-cause mortality (RR 0.73 and 95% CrI 0.42–1.23), cardiovascular mortality (RR 0.99 and 95% CrI 0.33–2.88), and repeat revascularization (RR 0.68 and 95% CrI 0.38–1.25) between these two treatments. The probability of optimal option for each strategy is presented in Figure 3. Angiography-guided CR was associated with the highest possibility of optimal therapy in terms of the primary outcome (98.5%), followed by FFR-guided CR (1.2%) and COR (0.3%). Angiography-guided CR was associated with the highest possibility of optimal therapy in terms of all-cause mortality (87.6%), MI (98.4%), cardiovascular mortality (50.4%), and repeat revascularization (90.7%). The inconsistency analysis demonstrated that the direct and indirect outcomes were consistent. The Bayesian meta-regression suggested that the variables had no correlation with the event rate of the primary outcome (Supplementary Table 1). The funnel plot indicated that no publication bias was found in the network meta-analysis (Supplementary Figure 5). The sensitivity analysis showed that the results were not influenced after excluding the unpublished study or using the odd ratio as effect size (Supplementary Table 2).

**Table 2:** The results of network meta-analysis.

**Outcome**	**RR (95% CrI)**	**Inconsistency** **analysis** **(*p*-value)**
**All-cause death or MI**		
Angiography-guided CR vs. COR	0.68(0.50–0.87)	0.564
FFR-guided CR vs. COR	1.06(0.75–1.66)	0.562
Angiography-guided CR vs. FFR-guided CR	0.64(0.38–0.94)	0.564
**All-cause mortality**		
Angiography-guided CR vs. COR	0.75(0.55–0.96)	0.284
FFR-guided CR vs. COR	1.03(0.62–1.69)	0.274
Angiography-guided CR vs. FFR-guided CR	0.73(0.42–1.23)	0.277
**Cardiovascular mortality**		
Angiography-guided CR vs. COR	0.68(0.38–1.01)	NA
FFR-guided CR vs. COR	0.68(0.25–1.71)	NA
Angiography-guided CR vs. FFR-guided CR	0.99(0.33–2.88)	NA
**MI**		
Angiography-guided CR vs. COR	0.63(0.43–0.86)	0.957
FFR-guided CR vs. COR	1.08(0.72–1.82)	0.954
Angiography-guided CR vs. FFR-guided CR	0.58(0.31–0.92)	0.970
**Repeat revascularization**		
Angiography-guided CR vs. COR	0.36(0.24–0.55)	0.876
FFR-guided CR vs. COR	0.53(0.32–0.87)	0.873
Angiography-guided CR vs. FFR-guided CR	0.68(0.38–1.25)	0.865

*MI, myocardial infarction; CR, complete revascularization; COR, culprit-only revascularization; CrI, credible interval; RR, relative risk; NA, not available*.

## Discussion

Our study had several findings: (1) angiography-guided CR indicated a lower incidence of primary outcome, all-cause mortality, and MI than COR, while the FFR-guided CR did not; (2) the angiography-guided CR showed a lower event rate of primary outcome and MI than FFR-guided CR; and (3) the angiography-guided CR showed the highest possibility of being the optimal CR strategy.

Research shows that the plaques of non-culprit vessel lesions in STEMI patients are vulnerable (37). The cardiac oxidative stress and inflammatory cytokine response after STEMI may lead to fibrous cap ruptures in the non-culprit lesions and cause new ischemia and MI events (20, 21). The development of new severe ischemic events or reinfarction may further expand the ischemic area, resulting in heart failure, malignant arrhythmia, and even death. Therefore, the prognosis of patients with STEMI combined with MVD is worse than that of patients with culprit vessels only (2, 38, 39). Previous observational studies have shown that CR could improve short- and long-term mortality in STEMI patients with MVD; however, these results may be biased by different factors (40). Several randomized controlled studies have been conducted recently, including the recently published COMPLETE study (5, 6, 34). Further, a meta-analysis suggested that traditional angiography-guided CR showed a lower incidence of adverse outcomes than COR (3, 4).

The FFR technology was used in interventions for stable MVD patients after its benefits were confirmed based on evidence. The results of the FAME study demonstrated that compared with angiography-guided CR, FFR-guided CR reduced the occurrence of the composite endpoint of mortality, MI, and revascularization (41). Several trials have evaluated the effectiveness of FFR-guided CR. In the COMPARE-ACUTE study, the FFR-guided CR indicated a lower event rate of composite outcome of death, recurrent MI, cerebrovascular events, and repeat revascularization than COR; however, the benefit of FFR-guided CR was driven by reduced incidence of repeat revascularization (6). Further, the results of the study by DANAMI-3-PRIMULTI were similar to those of the COMPARE-ACUTE study, which showed that FFR-guided CR did not reduce the incidence of adverse outcomes compared with COR (5). Therefore, the application of FFR-guided CR in STEMI patients with non-culprit vessel lesion remains controversial. Recently, the FLOWER-MI study was the only RCT to compare the effectiveness between FFR-guided and angiography-guided CR (8). The results suggested that compared with angiography-guided CR, FFR-guided CR had no benefit on further reduction of the composite outcomes of all-cause mortality and MI. Nevertheless, the FLOWER-MI study had limited power to assess the primary outcome. In this study, we conducted a Bayesian network meta-analysis to evaluate the efficacy of various CR strategies. Network meta-analysis can synthesize the effect size from direct and indirect comparisons to improve the accuracy of outcomes (9, 10). For instance, direct evidence comparing angiography-guided CR and COR suggested the corresponding 95% CI of the RR as 0.49–1.92, while the network meta-analysis indicated the corresponding 95% CrI as 0.36–0.99, which was narrower than that of direct evidence. Additionally, angiography-guided CR showed a lower incidence of adverse outcomes, such as all-cause mortality and MI, than COR, while FFR-guided CR did not. Angiography-guided CR had the highest possibility to be the optimal strategy. Therefore, angiography-guided CR was superior to FFR-guided CR in STEMI patients with obstructive non-culprit vessels.

The differences regarding adverse outcomes between angiography-guided and FFR-guided CRs could be explained by several factors. First, obstructive non-culprit lesions in STEMI patients are vulnerable. The COMPLETE substudy used optical coherence tomography to evaluate the association between the benefit of CR and the vulnerability of obstructive non-culprit lesions (42). The results showed that the obstructed non-culprit vessel had more thin-cap fibroatheromas than the non-obstructed vessel. Additionally, an obstructive thin-cap fibroatheroma had a greater mean lipid arc, higher lipid quadrants, and more cholesterol crystals and macrophages than an obstructive non-thin-cap fibroatheroma. Furthermore, in the FLOWER-MI study, the proportion of non-culprit lesions of 70–90% stenosis in the FFR-guided CR was lower than that in the angiography-guided CR (8). Therefore, patients in the FFR-guided CR group avoided premature intervention to the non-culprit vessel more frequently than those in the angiography-guided CR group, while most patients in the angiography-guided CR group completed CR to avoid the occurrence of cardiovascular events during the follow-up period. Additionally, the survival curves in the FLOWER-MI study gradually separated in the later stage of follow-up, which indicated that the non-culprit lesions in patients receiving the FFR-guided CR treatment became vulnerable as the follow-up period was extended (8). With the popularization of drug-eluting stents, optimization of the PCI technology, and iteration of antithrombotic drugs, the risk of non-culprit vessel intervention does not offset the benefits. Therefore, FFR-guided CR is not superior to angiography-guided CR.

### Comparison With Other Studies

Other meta-analyses have evaluated the effectiveness of CR in patients with STEMI and non-culprit vessel lesions. Ahmad et al. included 10 studies, with composite outcomes of cardiovascular death or MI as the primary endpoint, and found that compared with COR, the treatment of non-culprit vessel lesions could reduce the event rate of cardiovascular death and MI without reducing the risk of all-cause mortality (4). The meta-analysis by Pavasini et al. suggested that CR was associated with lower cardiovascular mortality than COR; however, this study was a pairwise meta-analysis only comparing the effectiveness of CR with COR without evaluating the influence of guiding technology on the clinical outcomes (43). The benefit of CR in reducing cardiovascular death has also been observed in a study by Bainey et al. (3) Additionally, the authors performed the subgroup analysis according to the type of guiding technology. The results showed that the angiography-guided CR was associated with a lower risk of cardiovascular death or new MI than COR, while the FFR-CR was not; however, the interaction analysis demonstrated no evidence of heterogeneity between these subgroups. Our study, using network meta-analysis to compare the effectiveness of different guiding technologies (FFR or angiography-guided) in the CR procedure suggested that the type of guiding technology was related to clinical benefits. Wald et al. developed a meta-analysis to evaluate the effectiveness between FFR-guided and angiography-guided CR (7). The results demonstrated that the angiography-guided CR showed a lower event rate of cardiovascular death and MI than COR, while FFR-guided CR did not. Additionally, all-cause mortality did not differ between CR and COR, which was not related to the guidance technology used. Our study is the first Bayesian network meta-analysis comparing effectiveness among various CR strategies. We found that angiography-guided CR showed a lower incidence of the primary outcome than FFR-guided CR. Therefore, CR should be performed under angiography guidance in clinical practice.

### Limitations of the Study

Our study has several limitations. First, the definition of MVD varied among the studies. For example, six studies defined MVD as an angiographic diameter stenosis >50% in more than one non-culprit artery (5, 6, 8, 28, 29, 31), while the other six studies defined MVD as stenosis diameter of >70% in more than one non-culprit artery (30, 32–36). Nevertheless, the result of a regression analysis showed that the stenosis of the non-culprit vessel had no correlation with the event rate of primary outcome. Second, there was only one study that compared the effectiveness of FFR-guided and angiography-guided PCI; thus, the comparison between these two revascularization strategies was mainly replied on the indirect evidence, which led to a limited power to evaluate the difference of effectiveness. For instance, the difference of the composite endpoint of angiography-guided CR vs. FFR-guided CR was mainly driven by the reduced MI risk, but the difference was with a large credible interval (CrI). Therefore, further studies directly comparing the effectiveness of these two CR strategies are warranted. Third, the results of the ISCHEMIA trial point to the fact that revascularization initially increases MI because it results in periprocedural MI (44). In the setting of STEMI, where cardiac markers are significantly elevated by default at baseline, any periprocedural MI as the result of CR of the remaining non-culprit lesions will largely be undetected and not accounted for. Our study suggested that the angiography-guided CR was superior to FFR-guided CR in reducing MI risk, in which the event rate may be underestimated. Therefore, further studies defining the outcome of MI in detail (especially the individual periprocedural MI) are warranted. Fourth, certain patients in the COMPLETE study received the FFR to identify the non-culprit vessel, but the outcome of these patients could not be obtained (8). Considering that the proportion of these patients was very small (<1%), we regarded patients in the CR group as those receiving angiography-guided PCI. Fifth, our study was mainly based on indirect comparisons (only one head-to-head study comparing angiography-guided CR and FFR-guided CR) and dependent on the choice of priors. Therefore, the results of our study should be interpreted cautiously. Sixth, there were few studies that reported the outcome of cardiovascular mortality; therefore, the statistical power was limited in the comparison between the angiography-guided CR and COR. Additionally, there was no direct comparison between angiography-guided CR and FFR-guided CR; we could not synthesize the direct and indirect evidence to compare the risk of cardiovascular mortality between angiography-guided CR and FFR-guided CR.

## Conclusions

Angiography-guided CR was superior to FFR-guided CR in patients with STEMI and MVD. However, only one study compared the effectiveness of FFR-guided and angiography-guided PCI; thus, the comparison between FFR-guided and angiography-guided PCI mainly relied on indirect evidence. Therefore, further studies directly comparing the effectiveness of these two CR strategies are warranted.

## Data Availability Statement

The original contributions presented in the study are included in the article/Supplementary Material, further inquiries can be directed to the corresponding author.

## Author Contributions

XC and LZ designed the study. WH and WG did the statistical analysis. LZ, WG, SH, XC, and CL wrote and revised the manuscript. LZ, WG, WH, LW, FM, XC, CL, and SH critically reviewed the manuscript. All authors contributed to the article and approved the submitted version.

## Funding

This study was supported by the Fund of Sanming Project of Medicine in Shenzhen (Nos. SZSM201603072 and SZSM201911019).

## Conflict of Interest

The authors declare that the research was conducted in the absence of any commercial or financial relationships that could be construed as a potential conflict of interest.

## Publisher's Note

All claims expressed in this article are solely those of the authors and do not necessarily represent those of their affiliated organizations, or those of the publisher, the editors and the reviewers. Any product that may be evaluated in this article, or claim that may be made by its manufacturer, is not guaranteed or endorsed by the publisher.
