# Supplementary material for: Comparative Effectiveness of Complete Revascularization Strategies in Patients With ST-Segment Elevation Myocardial Infarction and Multivessel Disease: A Bayesian Network Meta-Analysis
Source: Front Cardiovasc Med. 2021 Sep 23;8:724274. doi: 10.3389/fcvm.2021.724274 (PMC8496298; doi:10.3389/fcvm.2021.724274)
Supplement: Supplementary file 1 [file Data_Sheet_1.docx]

**Comparative Effectiveness of Complete Revascularization Strategies in Patients with ST-segment Elevation Myocardial Infarction and Multivessel Disease:**

**A Bayesian Network Meta-analysis**

**Supplemental Figure 1.** The flow chart of the research screening process.

**Supplemental Figure 2.** The assessment of the risk-of-bias of the included studies.

**Supplemental Figure 3.** Results of the meta-analysis for the comparison between fractional flow reserve-guided complete revascularization and culprit-only revascularization. FFR, fractional flow reserve; CR, complete revascularization.

**Supplemental Figure 4.** Results of the meta-analysis for the comparison between angiograph-guided complete revascularization and fractional flow reserve-guided complete revascularization. FFR, fractional flow reserve; CR, complete revascularization.

**Supplemental Figure 5.** The funnel plot of network meta-analysis. FFR, fractional flow reserve; CR, complete revascularization.

**Supplemental Table 1.** The results of Bayesian regression meta-analysis.

**Supplemental Table 2.** The sensitivity of network meta-analysis.

**Supplemental Figure 1. The flow chart of the research screening process.**


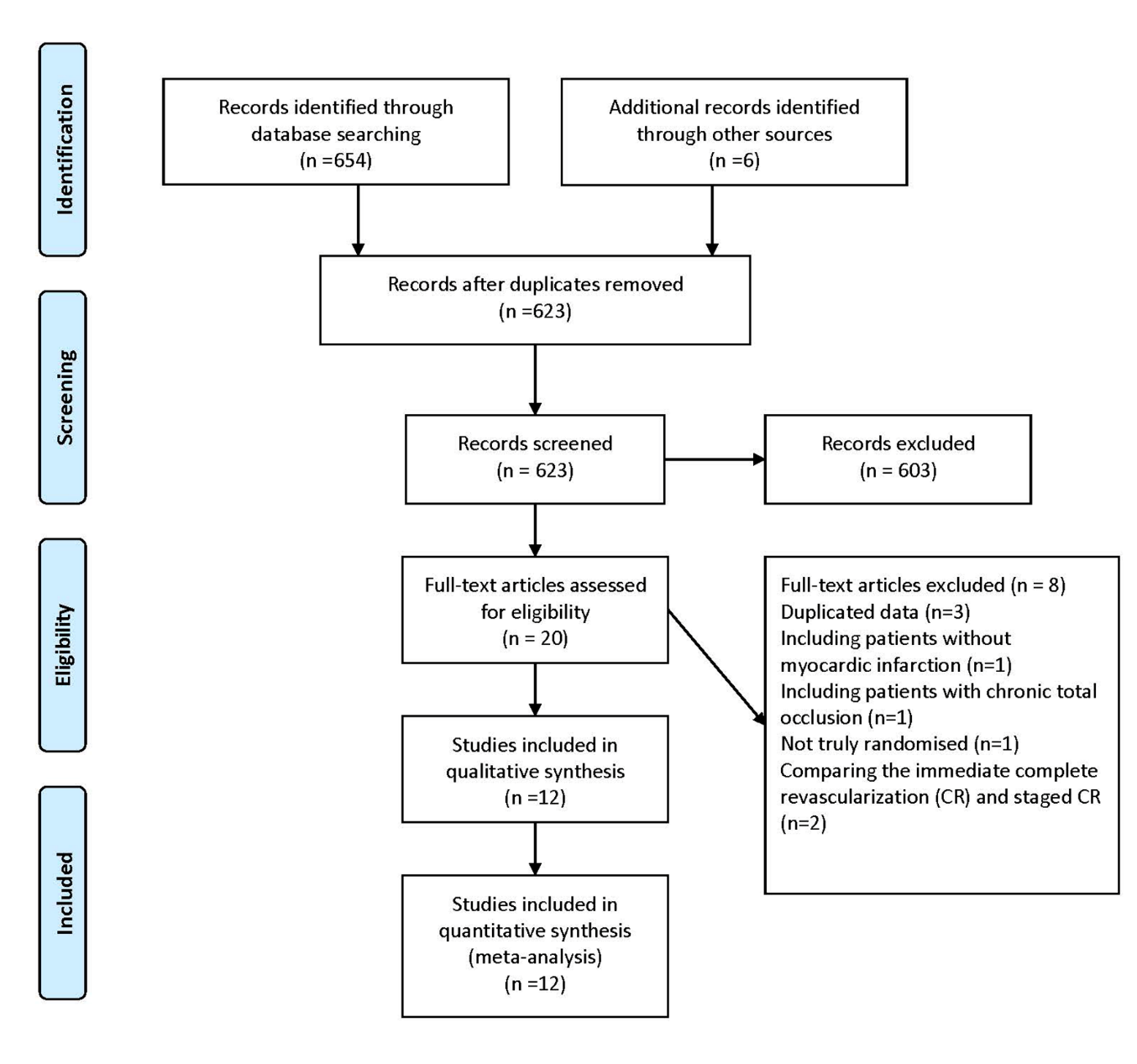


**Supplemental Figure 2. The assessment of the risk-of-bias of the included studies.**

**
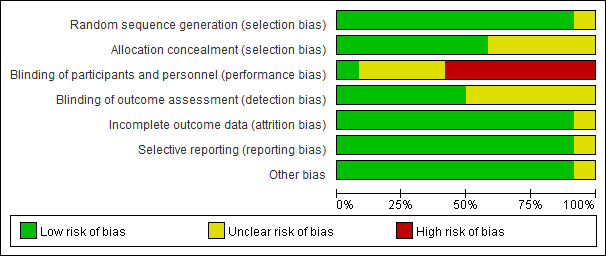
**

**
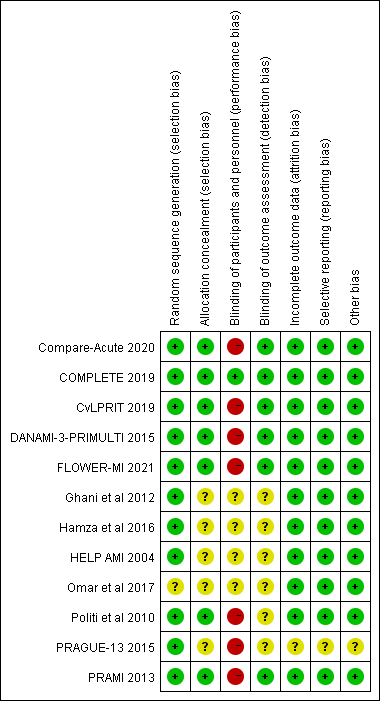
**

**Supplemental Figure 3. Results of the meta-analysis for the comparison between fractional flow reserve-guided complete revascularization and culprit-only revascularization.**

FFR, fractional flow reserve; CR, complete revascularization; COR, culprit-only revascularization


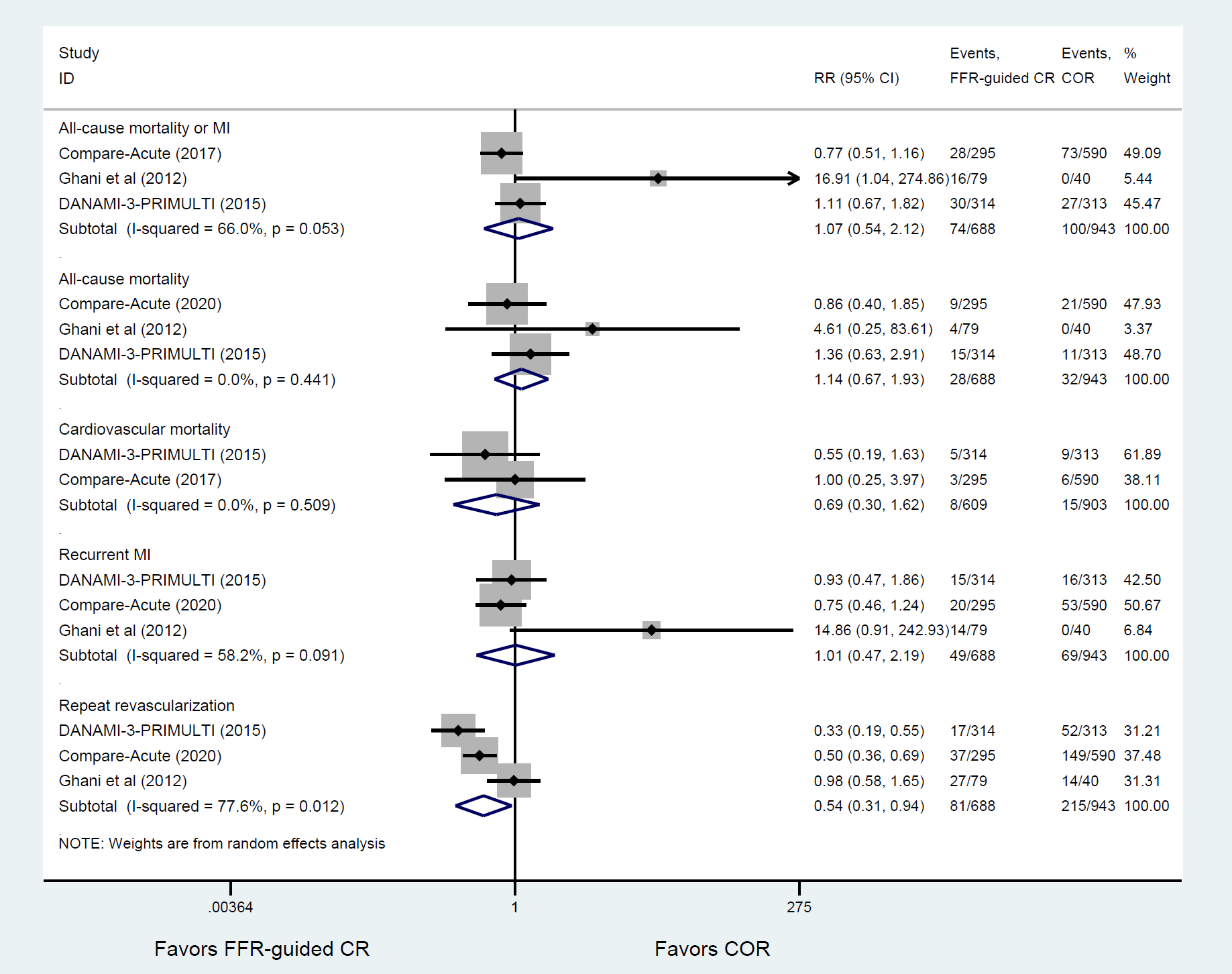


**Supplemental Figure 4. Results of the meta-analysis for the comparison between angiograph-guided complete revascularization and fractional flow reserve-guided complete revascularization.**

FFR, fractional flow reserve; CR, complete revascularization.

**
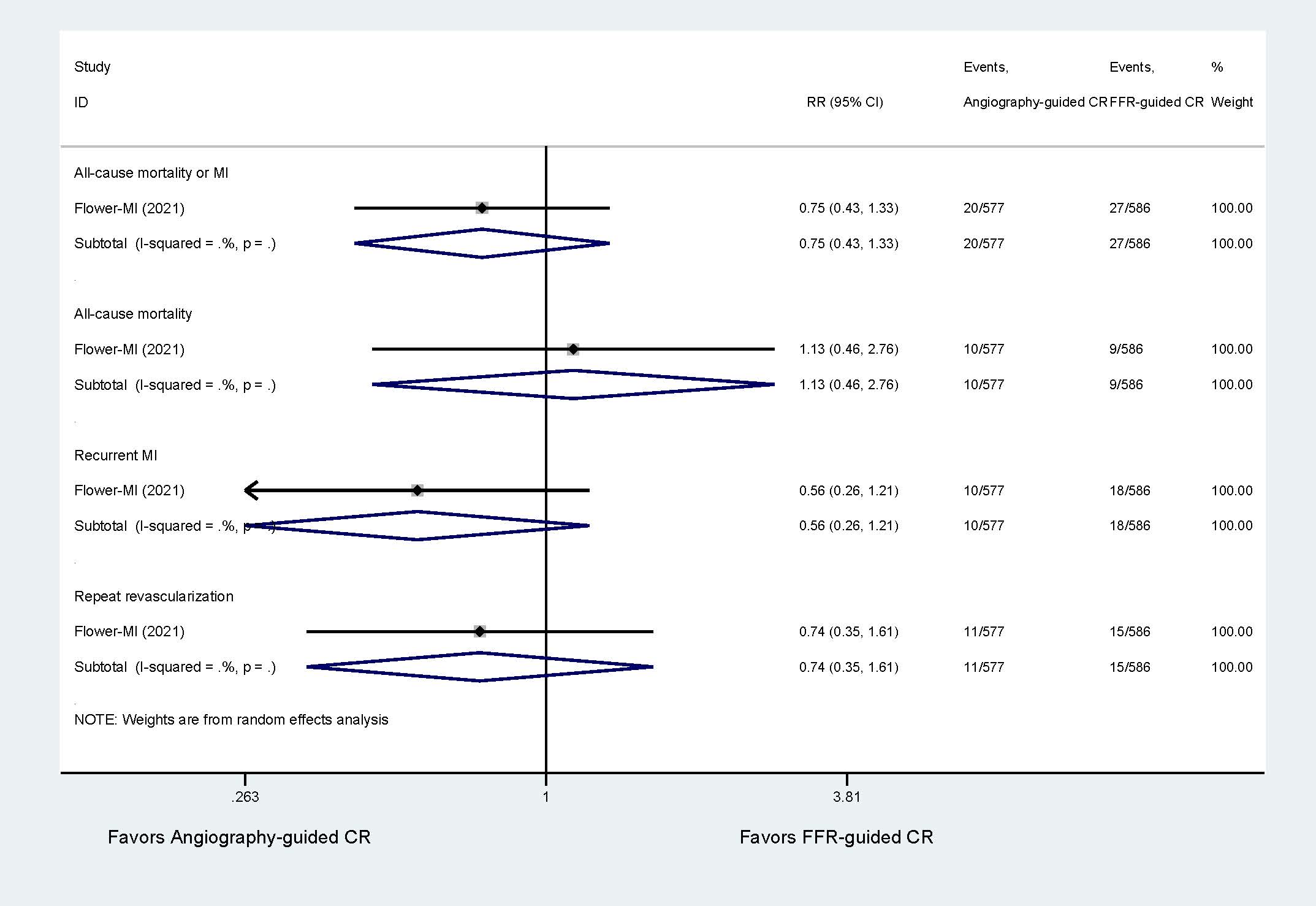
**

**Supplemental Figure 5. The** **funnel plot of network meta-analysis.**

FFR, fractional flow reserve; CR, complete revascularization; COR, culprit-only revascularization

**
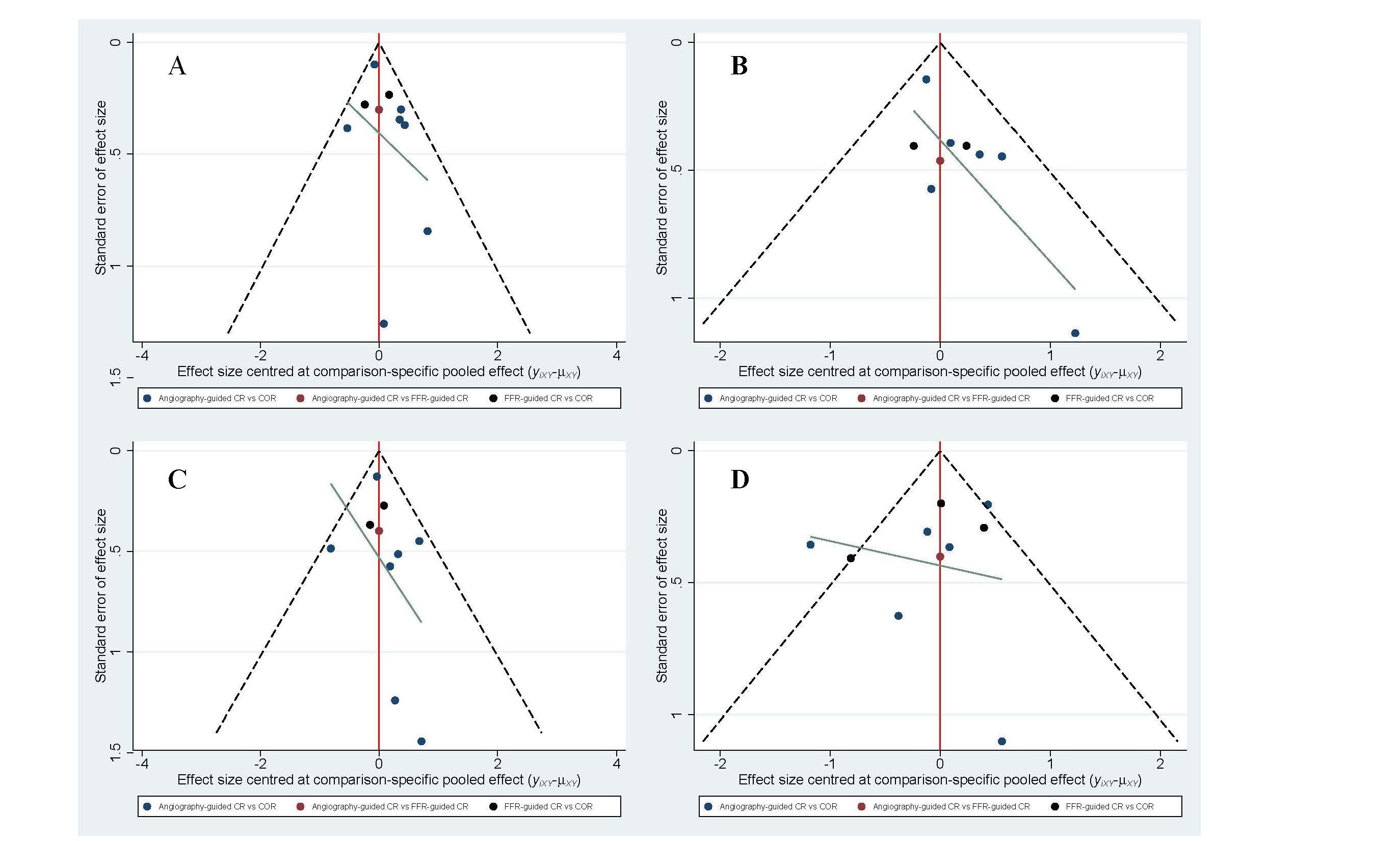
**

**Supplemental Table 1. The results of Bayesian regression meta-analysis.**

| **Variable** | **interaction term β** |
| --- | --- |
| Follow-up time | 0.19(-0.5—0.97) |
| Age | 0.22(-0.68—1.26) |
| The propotion of male | 0.3(-0.57—1.07) |
| The propotion of DM | -0.87(-2.19—0.21) |
| The propotion of anterior MI | -0.24(-1.17—0.68) |
| The stenosis of non-culprit  vessel lesson | 0.03(-0.76—0.68) |
| Three-Vessel Disease | -0.35(-0.9—0.21) |

DM, diabetes mellitus; MI, myocardial infarction.

| **Supplemental Table 2. The sensitivity of network meta-analysis.** | | |
| --- | --- | --- |
| **Comparison** | **Excluding the unpublished study** | **Using the Odd ratio as effect size** |
|  |  |  |
| Angiography-guided CR vs COR | 0.64(0.45-0.83) | 0.65(0.46-0.85) |
| FFR-guided CR vs COR | 1.05(0.74-1.62) | 1.05(0.72-1.71) |
| Angiography-guided CR vs FFR-guided CR | 0.61(0.35-0.90) | 0.62(0.34-0.93) |
| FFR, fractional flow reserve; CR, complete revascularization; COR, culprit-only revascularization | | |
